# Inhibition of oil digestion in Pickering emulsions stabilized by oxidized cellulose nanofibrils for low-calorie food design†

**DOI:** 10.1039/c9ra02417d

**Published:** 2019-05-14

**Authors:** Bin Liu, Yanli Zhu, Jingnan Tian, Tong Guan, Dan Li, Cheng Bao, Willem Norde, Pengcheng Wen, Yuan Li

**Affiliations:** College of Food Science and Engineering, Gansu Agricultural University Lanzhou 730070 China wenpch@126.com; Beijing Advanced Innovation Center for Food Nutrition and Human Health, College of Food Science and Nutritional Engineering, China Agricultural University Beijing 100083 China yuanli@cau.edu.cn; Physical Chemistry and Soft Matter, Wageningen University and Research Stippeneng, 4 6708WE Wageningen The Netherlands

## Abstract

Celluloses are renewable and biodegradable natural resources. The application of celluloses as oil-in-water Pickering emulsifiers is still quite limited. In this paper, cellulose nanofibrils (CNFs) with oxidation degrees (DOs) of 52.8% and 92.7% (DO50 and DO90) were obtained from TEMPO-mediate oxidation for microcrystalline cellulose (MC). The production of carboxyl groups of CNFs were confirmed by FT-IR and ^13^C solid-NMR. CNF-stabilized O/W Pickering emulsion showed excellent colloidal stability compared with un-oxidized cellulose by Turbiscan stability analysis. Additionally, CNF-stabilized Pickering emulsions showed stable colloidal properties in simulated intestinal fluid (SIF). Most importantly, *in vitro* fatty acid release kinetics under SIF showed that CNFs have strong inhibitory lipid digestion behavior. Our results suggest that the oxidation modification not only improves their emulsification activity but also promotes their application in oil digestion inhibition, providing inspiration for designing and developing low-calorie food products.

## Abstract 2

Oil digestion in CNF-stabilized Pickering emulsion was inhibited effectively.

## Graphical abstract

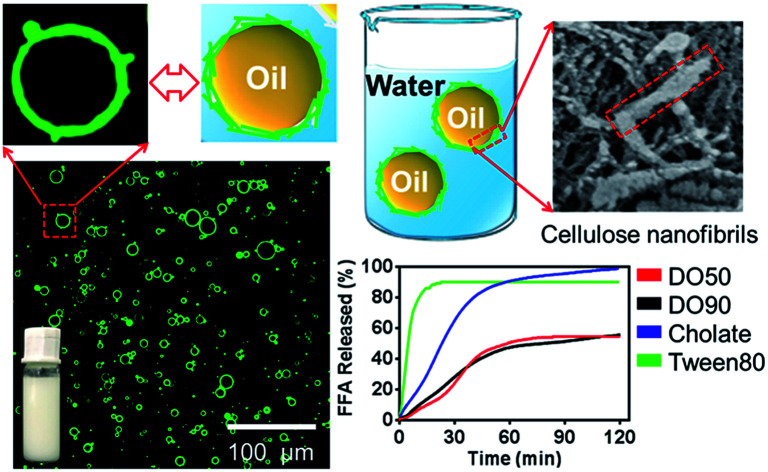

## 1 Introduction

Traditional emulsions are thermodynamically unstable systems containing two immiscible phases: the dispersed and continuous phase.^1^ A surfactant is necessary for stabilizing this unstable system. For instance, frequently used surfactants can be small molecules such as Tween-80 and Span-80,^2,3^ or macromolecular polymers, such as proteins and lipids.^4,5^ Compared with small molecular surfactants, solid colloidal particles can also be adsorbed at the oil–water interface and form “Pickering emulsions”. The solid particles are partially wetted by water and oil, and they could provide steric hindrance between the emulsion droplets. Pickering emulsions were first discovered by Ramsden^6^ and further developed systematically by Pickering later.^7^ These emulsions possess superior stability over traditional emulsions due to their extremely high desorption energy. Pickering emulsions are currently being used in petroleum industries,^8^ pharmaceuticals,^9^ nanoparticles synthesis,^10^ food applications,^11^ and other areas.

Recently, solid particles used to stabilize Pickering emulsions have included organic synthetic particles, such as graphene oxide,^12^ inorganic particles such as silica,^13^ and chemically modified starch.^14^ However, the lack of biodegradability and biocompatibility in Pickering emulsions stabilized by inorganic particles limit their application in food and pharmaceutical fields. For example, inedible or indigestible solid particles such as laponite, montmorillonite, and magnetic particles were difficult to apply in food systems.^15–18^ In recent years, scientists have paid much attention to the development of natural biopolymers for the preparation of food-grade Pickering emulsions. Some nanoparticles made of starch, soy protein, and chitosan were reported to stabilize Pickering emulsions.^19–21^ Li *et al.* prepared whey protein isolate (WPI) nanoparticles by heat cross-linking, which formed a stable Pickering emulsion when the pH was far from the isoelectric point (pI) of WPI.^22^ De Folter prepared a Pickering emulsion stabilized by zein nanoparticles which synthesized by an anti-solvent precipitation method, and they explored the effects of particle concentration, ionic strength and pH on the formation of stable Pickering emulsions.^23^ The plant protein isolated from quinoa crops formed a stable Pickering emulsion under ultrasonic treatment.^24^ Soybean-separated plant protein nanoparticles obtained from thermal treatment can form a stable Pickering emulsion *via* homogenization.^16^ Starch granules with varying properties were reported to form different Pickering emulsions under high-speed homogenization.^25^ Interestingly, stable Pickering emulsions with high mechanical interfacial membranes may delay fat digestion due to inhibited permeation of lipase to the inner oil phase. Sarkar *et al.* found that Pickering emulsions stabilized by soft whey protein microgel particles can effectively control the decomposition of emulsified fat by lipase.^26^ In addition, kafirin nanoparticle-stabilized Pickering emulsions can also delay fat digestion in simulated gastrointestinal (GI) conditions.^27^ However, these Pickering emulsions formed either by proteins or starch polysaccharides may be digested and unstable under gastrointestinal conditions. Additionally, the yield of Pickering particles of proteins and starches is quite low and their costs are quite high. Therefore, there is an urgent need to develop Pickering particulate emulsifiers that are more stable and cost-effective for food applications.

Because celluloses are non-digestible in the GI tract, cellulose nanoparticles-stabilized Pickering emulsions may be resistant and stable in the GI tract. Cellulose is the most abundant polysaccharide in nature and it is a renewable, biodegradable, and underutilized resource. Celluloses are composed of d-glucopyranose rings that are linked by a β-(1,4)-glycosidic bond. They were insoluble and have a high molecular weight, which greatly limits their application as emulsifiers. Therefore, improving their aqueous dispersibility and down-scaling their size by structural modification may improve their aqueous wetting and emulsification properties. Most current modification methods lack selectivity of reaction sites, and the randomness of reaction results in a low degree of oxidation which limited the dispersibility of cellulose. It has been reported that TEMPO (2,2,6,6-tetramethyl-1-piperidinyloxy) mediated oxidation can selectively oxidize C6 primary hydroxyl groups of polysaccharides in aqueous dispersion medium, which has the advantages of high selectivity and controllable oxidation degree.^28,29^ It has been widely used for oxidation of polysaccharides, including starch and lignin.^30,31^ Isogai *et al.* studied the oxidative properties of the TEMPO/NaClO/NaBr oxidation system for various cellulosic materials. Mercerized cellulose or regenerated cellulose rather than natural cellulose with high crystallinity could be oxidized completely.^32^ Currently, TEMPO-mediated oxidation of celluloses has potential applications mostly in high gas-barrier films for packaging and display, fine separation filters and health care materials.^33^ Bryan *et al.* proved that TEMPO-mediated oxidation of celluloses have low toxicity to developing zebrafish.^34^ However, celluloses for preparing oil digestion inhibited Pickering emulsions are less understood. Herein, we attempt to chemically modify tree-derived MC by TEMPO/NaClO/NaBr oxidation system in order to obtain aqueous-dispersible cellulose nanofibrils (CNFs). The aim of this study was to design CNF-stabilized Pickering emulsions with inhibited oil digestion. First, the physiochemical properties of CNF were measured. Then, the CNF-stabilized oil-in-water (O/W) Pickering emulsions were characterized. Next, the oil digestion ability of CNF-stabilized Pickering emulsion was investigated under simulated gastrointestinal fluid compared with Tween 80 and cholate-stabilized emulsions. Finally, the stability of the Pickering emulsion was determined by Turbiscan stability analyzer. Our results showed that CNFs have great potential for stabilization of Pickering emulsions by slowing down oil digestion.

## 2 Materials and methods

### 2.1 Materials

Microcrystalline cellulose (MC) was purchased from Aladdin Bio-Chem Technology Co., Ltd (Shanghai, China). TEMPO, Super Green-1 and Nile Red were purchased from Sigma-Aldrich. Pepsin, lipase and bile salts of pig were purchased from Biosharp (Wuhan, China), pancreatin was purchased from Aoboxing Biotechnology Co., Ltd (Beijing, China), and palm fruit oil was from COFCO Corporation (Beijing, China). All other chemicals with analytical grade were obtained from Beijing Chemical Factory (Beijing, China). All chemicals without any further purification were used. Ultra-pure water was prepared *via* a Millipore Super Q apparatus.

### 2.2 Preparation of TEMPO-mediated oxidized CNFs

TEMPO-mediated oxidized CNFs were prepared according to previous studies.^35^ Briefly, 2 g MC was dispersed in water, followed by the addition of 0.013 g TEMPO and 0.05 g sodium bromide, and adjusted to pH 10. NaClO (10%, pH = 10) and NaOH (0.5 M) solution was gradually added dropwise to maintain the pH during the reaction until the volume of NaOH solution was required for corresponding degree of oxidation (DO) of CNFs. In this study, CNFs with theoretical DOs of 30%, 50% and 90% (defined as DO30, DO50 and DO90) were prepared by controlling the consumption of NaOH volume. Particularly, a completely oxidized DO100 CNF was prepared by adding an excess of NaClO to achieve complete oxidation and actual DO100. To terminate the reaction, 2 mL ethanol was added. Next, 0.05 g sodium borohydride was added and stirred for 1 h to fully reduce the ketone groups. The solution was adjusted to pH 3 and maintained for 1 h, then adjusted back to pH 7 at a same time interval. Finally, equivalent volume of ethanol was added to the above solution and stirred for 1 h. The final product was harvested by suction filtration and washed with ethanol three times. The obtained precipitate was extensively dialyzed against water for 48 h to remove any remaining contaminants, and the solution was lyophilized to obtain the products.

### 2.3 Proton titration of the CNFs

The charge density of the CNFs (theoretical DO30, DO50, DO90, and complete DO100) was determined by proton titration (Mettler Toledo, T50, Switzerland) referred to Li *et al.* with some modifications.^36^ In detail, the titration chamber was evacuated with argon and maintained at 25 °C. 0.1 M NaOH and HCl were used. 50 mL CNF (0.4 g L^−1^) was dissolved in 0.01 M NaNO_3_ aqueous solution. The pH of the system was first adjusted to 3 and stand for 30 min. Then, the titrations were carried out by titrating the sample back to 9.0. The charge density (C g^−1^) of the CNF was calculated based on the amount of NaOH and HCl used in the process. Then the titration curve of charge density *vs.* pH can be plotted. The maximum charge density can be obtained from the plateau value of the titration curves.

### 2.4 Preparation of oil in water (O/W) Pickering emulsions

The Pickering emulsion was prepared using ultrasonic emulsification method *via* an ultrasonic cracker (Sonics, VC800, USA). Dispersed phase is palm fruit oil and the continuous phase is 0.1% (wt) aqueous suspension of MC, DO50, and DO90 respectively. The oil to water ratio is 1/10 (v/v). According to Hattrem *et al.*,^37^ size of the emulsion droplet was monitored by Malvern Mastersizer 3000 (Malvern, UK) connected to a wet dispersion unit (Hydro EV, Malvern, UK) and the data was recorded using the respective software (Mastersizer 3000, v1.0.1). Microscopic morphology of emulsion droplets was observed by an optical microscope (UOP, UB103i, China). Pickering emulsion stained with Super Green-1 or Nile Red was observed by a laser scanning confocal microscope (Leica, SP2, Germany).

### 2.5 Stability analysis of O/W Pickering emulsion

The stability analyser Turbiscan applies the principle of multiple light scattering. The intensity of transmitted and backscattered light from the detector is determined directly by the concentration (volume percentage) and average diameter (or the average diameter of particles/droplets) of the dispersed phase.^38^ By measuring changes in transmitted light and backscattering intensity, it is possible to know the change in the concentration or particle size of a sample. In short, the relative stability of each system can be compared using the Turbiscan stability index (TSI) as shown below.1
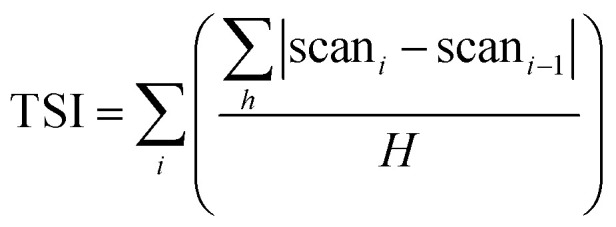
‘*i*’ is the number of measurements; ‘*h*’ is the height of the instrument scan; ‘scan’ is backscattered light or transmitted light intensity; ‘*H*’ is the measured maximum height.

The prepared Pickering emulsions in PBS of pH 7, ionic strength of 150 mM were placed in the incubator of the Turbiscan analyser (Formulaction, Tower, France) and the temperature was controlled at 25.0 ± 0.5 °C. The particle size change was measured within 24 h to analyse the stability of the emulsions.

### 2.6 *In vitro* digestion of lipid (palm fruit oil) encapsulated in the Pickering emulsion

*In vitro* digestion of Pickering emulsion was measured by automatic potentiometric titration (Metrohm, 907 Titrando, Switzerland). In detail, 5 mL of bile salt solution, 1 mL of CaCl_2_ solution (60 mg mL^−1^), 1.5 mL of lipase solution (60 mg solution, 1.5 mL PBS) were added to 30 mL Pickering emulsion. After the digestion procedure was completed, the released free fatty acids (FFA) during the *in vitro* digestion were reflected by comparing the volume and rate of consumption of NaOH. Experimental Pickering emulsions were made of CNFs with DO50 and DO90, and the emulsions formed by Tween 80 and bile salts, served as control. The released FFA was calculated by the equation below:^39^2
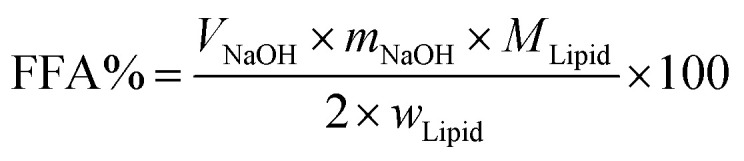
Here *V*_NaOH_ is the volume of sodium hydroxide required to neutralize the FFA produced (L), *m*_NaOH_ is the molarity of the sodium hydroxide solution used (in M), *w*_Lipid_ is the total mass of triacylglycerol oil initially present in the digestion cell (in g), and *M*_Lipid_ is the molecular mass of the palm fruit oil (0.88 g mol^−1^).

## 3 Results and discussion

### 3.1 Preparation and characterization of TEMPO oxidized cellulose nanofibrils

**Fig. 1:**
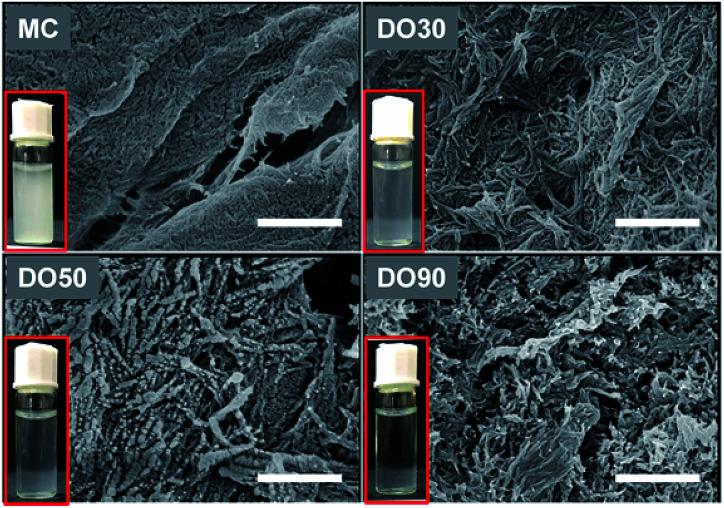
SEM image of MC, DO30, DO50 and DO90. And their aqueous dispersability (inset, 1 mg mL^−1^). The scale bar is 500 nm. SEM (JEOL, JSM 6301F) with a 5 mm working distance and an acceleration voltage of 5 kV.

Oxidation technique is widely used in the chemical modification of polysaccharides, such as starch and konjac glucomannan to provide them with charged and functional groups.^40,41^ Cellulose is insoluble in water, but can receive a large amount of carboxyl groups after oxidation to improve its water dispersibility. During TEMPO oxidation, C6 hydroxyl groups of cellulose are selectively oxidized into carboxyl ones, while the microcrystalized structure is disintegrated into nanocrystals. Thus, well-dispersed CNFs can be obtained. We prepared various oxidized celluloses with different degree of oxidation (DO) and their micromorphology and physicochemical properties were characterized. Fig. 1 illustrates of the morphology of MC and TEMPO-oxidized MC with a theoretical DO of 30%, 50% and 90% (referred to as DO30, DO50 and DO90) by SEM. The un-oxidized MC is densely structured; after oxidation, nanosized fibril-like CNFs are obtained, and the fiber structure becomes more and more open as the DO increased. In addition, the cellulose particle size has dropped from the original 25 μm to a few hundreds nanometers (ESI Fig. S1†). During the TEMPO oxidation, the primary alcohol groups on the glucose unit on cellulose are converted into carboxyl groups. The repulsion between the charged chains may greatly weaken the hydrogen bonding between the cellulose chains and destroys their highly crystallized regions. Therefore, CNFs were produced after TEMPO oxidization. The higher the DO, the more crystallization zones are destroyed and the less dense the structure is. The turbidity experiments in ESI Fig. S2† demonstrates the aqueous dispersibility changes of MC before and after oxidation with different DO. It can be seen that the un-oxidized MC is insoluble in water with a high turbidity at 600 nm, forming a milky white suspension. While the low turbidity of CNFs indicated that the water dispersibility of DO30, DO50 and DO90 was improved significantly, and the water dispersibility increases with increasing DO. Moreover, the dispersity of the synthesized CNFs in water can be directly recognized by Tyndall phenomenon again indicating improved aqueous dispersibility (ESI Fig. S3†). On the one hand, TEMPO oxidation alters the micro-structure of MC, disintegrating the highly crystalized structure into dispersible segments such as CNFs. On the other hand, more hydrophilic carboxyl groups introduced with increasing DO, resulting in a rise of negative charges (Table 1), and the enhanced electrostatic repulsion between the CNFs chains.

**Table 1:** Zeta-potentials of MC, DO30, DO50, and DO90 polymers

Sample	Zeta-potential (mV)
MC	−2.6 ± 0.4
DO30	−25.8 ± 0.9
DO50	−50.8 ± 0.5
DO90	−59.4 ± 1.0

**Fig. 2:**
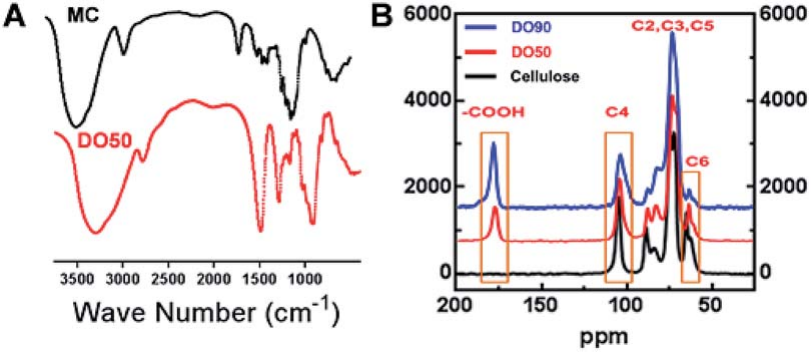
Characterization of TEMPO oxidized cellulose nanofibrils. (A) FT-IR spectra of MC that before and after TEMPO-mediated oxidation with an infrared region of 4000 to 500 cm^−1^. (B) ^13^C solid-NMR spectra of TEMPO-oxidized cellulose polymer with various degree of oxidation (DO50 and DO90). Operation conditions: spinning rate 5 kHz, pulse delay 5 s and contact time 5 ms.

As illustrated in the FT-IR spectra (Fig. 2A), the symmetric stretching vibration (*ν*_s_) at 1600 cm^−1^ and 1390 cm^−1^ are the characteristic absorption peaks of carboxyl groups.^42^ This finding indicates that the carboxyl group was truly introduced into the CNFs after the TEMPO oxidation. In addition, the ^13^C solid-state NMR spectra further demonstrate the exact carbon position of the glucose unit on cellulose under TEMPO-mediated oxidation. As shown in Fig. 2B, a new absorption peak appeared at ∼175 ppm in both DO50 and DO90, which are the characteristic peaks of the carboxyl group.^43^ The peak area of C6 gradually decreases with increasing DO while the peak area at ∼175 ppm increases with increasing DO, indicating that C6 alcohols were successfully oxidized into carboxyl groups. According to literature, the disappearance of the C6 carbon resonances at 61–62 ppm in ^13^C NMR spectrum indicates that the primary alcohol groups are completely oxidized.^44^ Therefore, it can be concluded from ESI Fig. S4,† the disappearance of the C6 carbon resonances indicating that the oxidation degree of complete oxidized DO100 is indeed 100%, which can be defined as a standard to calculate the experimental DO for theoretical DO30, DO50, and DO90. In addition, the peak areas remain essentially unchanged at the C2, C3, C4, and C5 positions. Some literature reported that ketones may occur when the secondary hydroxyl groups at the C2 or C3 positions are oxidized by the TEMPO-mediated oxidation.^32^ However, our oxidized celluloses have no absorption peaks at the corresponding resonance signal of 220–210 ppm for ketones, indicating no oxidation of secondary hydroxyl groups occurred (ESI Fig. S5†). This demonstrated that TEMPO-mediated oxidation specifically occurred at C6 position. These data are in accordance with the chemical structure variation of other polysaccharides after TEMPO-mediated oxidation.^45,46^ Finally, the maximum surface charge of each CNF (theoretical DO30, D050, DO90, and completely oxidized DO100) was obtained *via* proton titration (Table 2). The actual DO for theoretical DO30, DO50, and DO90 calculated from the ratio of their surface charges dividing by that of complete DO100 were 31.2%, 52.8% and 92.7%, respectively. The combination of FT-IR, ^13^C solid-state NMR and proton titration results demonstrated that CNFs with various degree of oxidation were successfully prepared.

**Table 2:** The maximum charge density and actual DOs of various CNFs

Theoretical DO	Maximum charge density (C g^−1^)	*R* a	Actual DO/%	Defined as
30	−61.43	0.312	31.2	DO30
50	−103.86	0.528	52.8	DO50
90	−182.37	0.927	92.7	DO90
Complete 100	−196.71	1	100	Standard/DO100

a *R*: the ratio of surface charges of oxidized celluloses (theoretical DO30, DO50, and DO90) to the completely oxidized DO100.

### 3.2 Preparation and characterization of cellulose nanofibrils stabilized Pickering emulsion

**Fig. 3:**
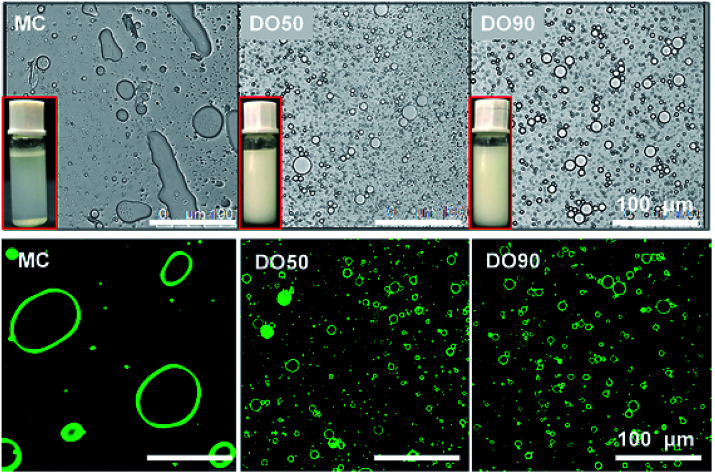
The micrograph and CLSM image of Pickering emulsions stabilized by MC, DO50 and DO90 at room temperature shooting by optical microscope and fluorescence confocal microscope, separately. The excitation and emission wavelength of Rhodamine B is 543 nm and 625 nm, respectively. The inset shows a digital photograph of the corresponding emulsions. The oil to water ratio is 1/10 (v/v).

The Pickering emulsions are prepared by ultrasonic emulsification at an oil/water volume ratio of 1 : 10. Here we used 10% fat content emulsion as the normal oil content in common foods rather than high calorie foods to study the slowed fat digestion effect, because commonly used oil content can represent most food systems. As shown in Fig. 3, it was clearly seen that MC cannot stabilize and form an emulsion, while both DO50 and DO90 can form a well droplet-dispersed O/W emulsion. Microscopic images of those emulsions further showed that MC do not form emulsion droplets while DO50 and DO90 CNFs have many emulsion droplets distributed in solution (Fig. 3, upper). The droplet sizes for DO50 and DO90 stabilized emulsions were around 15 μm, which were larger than Tween 80 emulsions (11 μm) and smaller than cholate emulsions (18 μm) (ESI Fig. S6†). The large size of MC-emulsions (∼100 μm) may be due to the aggregation of emulsion droplets. To further verify that the CNFs are adsorbed at the oil–water interface, positively charged fluorescent dye Super Green-1 was applied to label the negatively charged DO50 and DO90. CNFs stabilized oil/water Pickering emulsions were then visualized by confocal laser scanning microscope (CLSM). A homogeneous distribution (green ring) of DO50 and DO90 at the interfaces suggest a solid evidence for the Pickering emulsions stabilized by CNFs (Fig. 3, lower). This result is similar to that found by Wu *et al.*^22^ Apparently, the MC could not make stable Pickering emulsion may be due to its overlarge particle size of 25 μm. Because it is reported that only moderately degraded microfibrils (5–10 μm long) could stabilize oil in water emulsions.^47,48^ In order to better control the emulsion stability, the particle size for stabilizing the Pickering emulsions must be further reduced. In addition, an increase in carboxyl groups after TEMPO-mediated oxidation increased their hydrophilicity, which increased the dispersibility of DO50 and DO90 in the aqueous continuous phase, favoring the stabilization of the O/W Pickering emulsion.

### 3.3 Inhibition effect of lipid digestion by CNFs stabilized Pickering emulsions

**Fig. 4:**
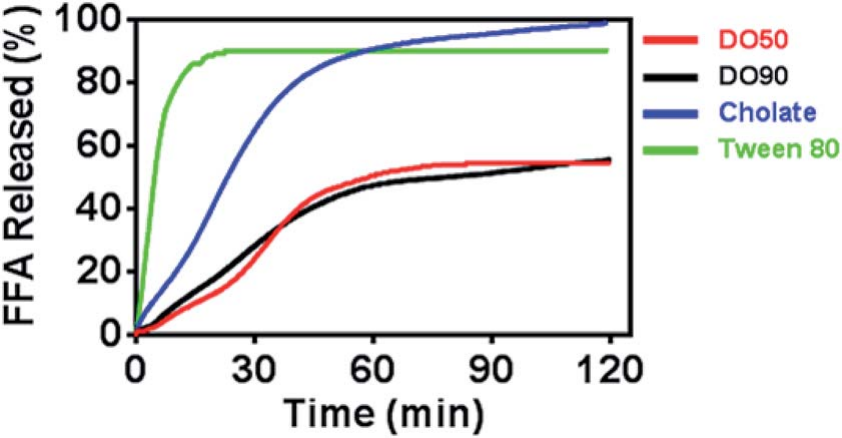
Time dependence of FFA release from Pickering emulsions stabilized by DO50, DO90, cholate and Tween 80. The oil to water ratio is 1/10 (v/v).

Oil droplets provide important texture and sensory perception for foods. However, the oil contained in emulsion droplets in food may increase their calorie count, which is considered to be unhealthy. The inhibited oil digestion in food emulsions may be beneficial for decreased calorie intake and retaining their texture and flavours. To determine whether CNF-stabilized Pickering emulsions can slow down oil digestion, the release kinetics of FFAs of various O/W emulsions under simulated intestinal fluid containing pancreatic lipase were measured. The oil phase contained equal amount of triacyclglycerols (TG), and CNF-stabilized emulsions were compared with emulsions stabilized by cholate (native emulsifier in human body) and Tween-80 (commercial emulsifier) during 2 h of lipase hydrolysis by a pH-stat titrator (Fig. 4). 10% TG in emulsions represents the normal fat content in common foods. The results showed a distinctly rapid release of FFA to 90% and then reached a pseudo-plateau value by Tween-80 stabilized O/W emulsions at 20 min. The cholate stabilized O/W emulsions reached 90% FFA at 60 min. For the DO50 and DO90 group, less than 50% FFA was released within 2 hours, achieving a slowed FFA release profile, indicating that CNF-stabilized Pickering emulsions indeed have an inhibitory effect on TG digestion. The interfacial structures of these emulsions are different which may further influence their oil digestion. The small surfactant Tween 80 may form a very thin monolayer around the emulsion droplets, so lipase may easily approach to the oil phase. Indeed Tween 80 stabilized emulsion showed the fastest oil digestion kinetics, while CNFs may form a dense network at the interface that prevents the approach of the lipase to the inner oil phase. Similarly, Kargar *et al.* also discovered a thick layer of microcrystalline cellulose on the emulsion interfaces which significantly reduced the lipid oxidation rate.^49^ Additionally, celluloses are capable of binding TG molecules, which may inhibit lipase hydrolysis.^50^ The TG combined cellulose complexes later enter the colon for fermentation or excretion. Furthermore, cellulose is also considered as a healthy ingredients working as prebiotics beneficial for probiotics proliferation.^51^ Therefore, CNFs show great potential serving as low calorie and health-promoting Pickering emulsifiers that can be applied in emulsion preparation for food industry.

### 3.4 Stability of DO50 stabilized Pickering emulsion in simulated gastrointestinal fluids

**Fig. 5:**
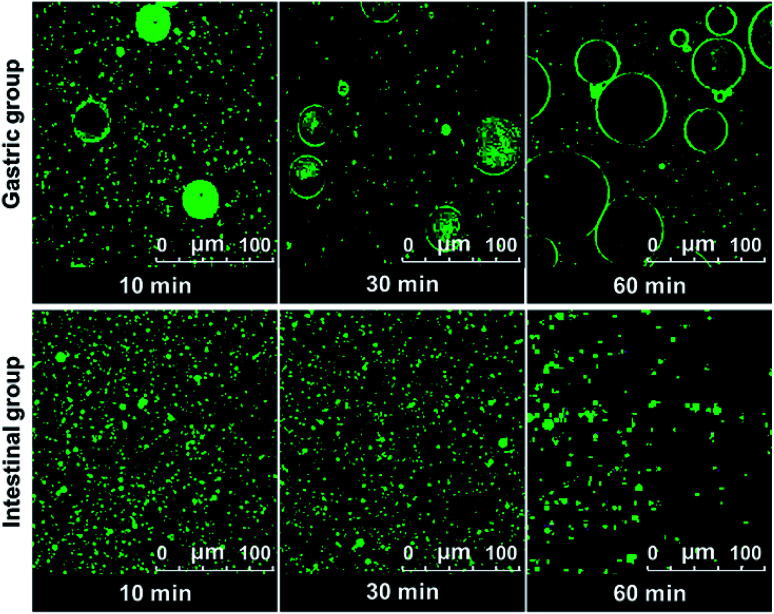
CLSM images of Pickering emulsion formed by DO50 digested in the simulated gastric (gastric group) and intestinal fluid (intestinal group) at 37 °C during 1 h. The oil to water ratio is 1/10 (v/v). The excitation and emission wavelength of Rhodamine B is 543 nm and 625 nm, respectively.

The digestion of CNF-stabilized Pickering emulsion is important for using it in food application. It is beneficial for us to further develop practical food products by exploring the stability of Pickering emulsions during the digestion process. The stability of emulsion in the gastrointestinal (GI) tract can influence the digestion of oil in emulsions. Therefore, the stability of CNF-stabilized Pickering emulsions in simulated GI conditions was investigated. DO50 was selected to further study their stability in gastrointestinal fluid. Changes of DO50 stabilized Pickering emulsions in simulated GI fluids were studied. The stability of fluorescently labelled DO50 Pickering emulsions in simulated GI fluid was observed by CLSM (Fig. 5). DO50 stabilized Pickering emulsions were found to be quite stable at intestinal conditions, while they were coalescent at stomach conditions. In gastric conditions, Ostwald ripening may occur, where small emulsion droplets gradually merge into many larger ones.^52^ This may be due to that DO50 is protonated by plenty of H^+^ contained in the simulated gastric fluid, resulting in a decrease in the surface charge and screening of the repulsive force between the droplets. In contrast, in alkaline intestinal fluid, the dissociation of carboxyl groups on DO50 was promoted, which increased their surface charges and repulsion, and therefore, stabilized the emulsion droplets. However, the decreased fluorescence intensity in simulated intestinal conditions probably because a small portion of the oil contained in the emulsion droplets was degraded by pancreatic lipase and caused demulsification. For control groups of Tween 80 and cholate stabilized emulsions (Fig. S7†), Tween 80-emulsions contained relatively small droplets that were evenly distributed during gastric digestion. However, in simulated intestinal fluid conditions, it was digested so fast that there was almost no fluorescence signal observed after 10 min. Cholate-emulsions were almost completely digested in simulated intestinal fluid after 30 minutes. These results were consistent with the release of FFA, which suggested that the CNF-stabilized Pickering emulsions were rather stable at gastrointestinal digestion process.

### 3.5 Stability analysis of O/W Pickering emulsion by Turbiscan

**Fig. 6:**
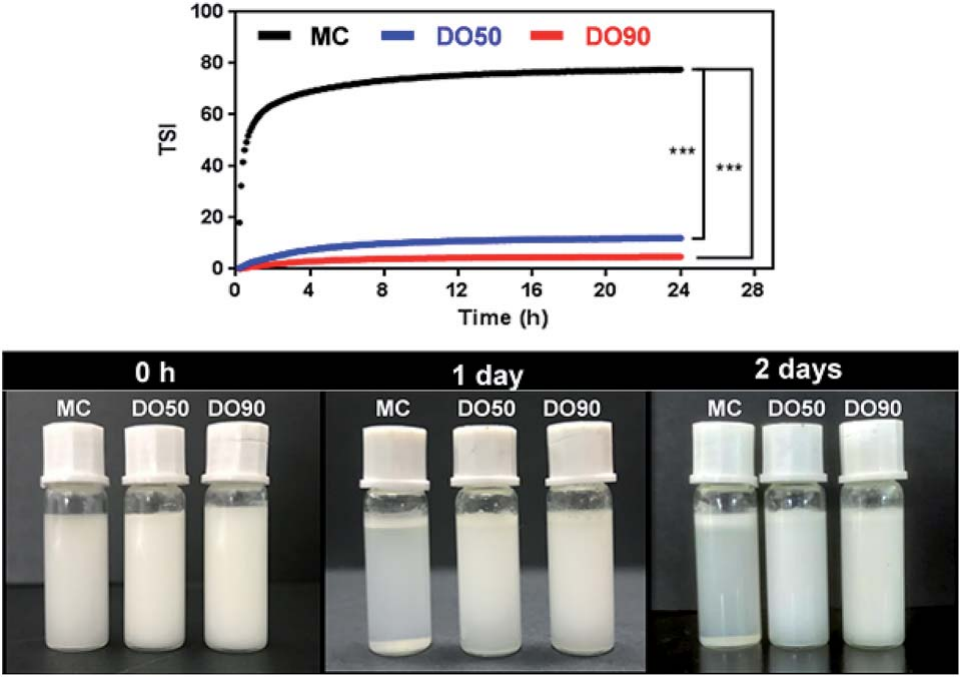
Kinetic instability diagram (upper) and pictures (down) of MC, DO50 and DO90 stabilized Pickering emulsion in 24 h. Experiments were performed at least triplicate and no significant difference in the same sample (*P* > 0.05). *p < 0.05, **p < 0.01. ***p < 0.001. The prepared Pickering emulsions (oil to water ratio is 1/10 (v/v)) in PBS of pH 7, ionic strength of 150 mM and the temperature was controlled at 25.0 ± 0.5 °C.

TSI can be calculated from the changes in transmitted light and backscattering intensity of liquid sample by the Turbiscan analyzer, which can show the change in the concentration or particle size of a sample during storage. Larger TSI values reflect less system stability. The TSI of MC-stabilized Pickering emulsion increased from 0 to 75 within 1 h, while the DO50 and DO90 maintained a relatively low value of 10 and 5 for up to 24 hours (Fig. 6). The results quantitatively indicate the order of colloidal stability of these three emulsions: DO90 ≈ DO50 ≫ MC. Emulsion formed by MC went through a creaming quickly, however, the emulsions stabilized by DO50 and DO90 did not cream for two days, indicating an excellent storage stability of CNFs stabilized emulsions. This may be due to the reduced particle size of the oxidized CNFs and a certain amount of negative charges, both of which were beneficial to form a stable Pickering emulsion. The reduced particle size allows DO50 or DO90 to be more easily adsorbed on the oil–water interface, thereby facilitating oil in water emulsion formation. The charge may increase the repulsion between emulsion droplets, which avoiding the aggregation and Ostwald ripening of the droplets. Finally, emulsion creaming was prevented. Furthermore, DO50 and 90 can form a dense 3D network film on the surface of emulsion droplets, it forms a barrier between emulsions and effectively preventing droplets aggregation.^53^ These stabilization mechanisms not only benefit the stability of the food processing process but also facilitate storage stability and extend their shelf life.^54^

## 4 Conclusions

In summary, CNFs with different DOs were successfully obtained from TEMPO-mediated oxidation for tree-derived MC. Colloidal stable Pickering emulsions were produced by DO50 and DO90 *via* ultrasonic emulsification. CNFs had stable colloidal properties at simulated intestinal conditions. Most importantly, CNF-stabilized Pickering emulsions exhibited a strong inhibitory effect on TG digestion compared with cholate and Tween 80. CNFs show great potential as health-promoting particulate emulsifiers that can be applied in designing low-calorie foods.

## Conflicts of interest

There are no conflicts to declare.

## Supplementary Material

RA-009-C9RA02417D-s001
