# Supplementary material for: Inhibition of oil digestion in Pickering emulsions stabilized by oxidized cellulose nanofibrils for low-calorie food design
Source: RSC Adv. 2019 May 14;9(26):14966–73. doi: 10.1039/c9ra02417d (PMC9064222; doi:10.1039/c9ra02417d)
Supplement: RA-009-C9RA02417D-s001 [file RA-009-C9RA02417D-s001.pdf]

Electronic Supplementary Material (ESI) for RSC Advances.
This journal is © The Royal Society of Chemistry 2019

Supplementary Information for:

Inhibition of oil digestion in Pickering emulsions stabilized by oxidized cellulose nanofibrils for low-calorie food design

Bin Liu,[ab] Yanli Zhu,[a] Jingnan Tian,[b] Tong Guan,[b] Dan Li,[b] Cheng Bao,[b] Willem Norde,[c] Pengcheng Wen[a] and Yuan Li*[b]*

[a]College of Food Science and Engineering, Gansu Agricultural University, Lanzhou 730070, China

[b]Beijing Advanced Innovation Center for Food Nutrition and Human Health, College of Food Science and Nutritional Engineering, China Agricultural University, Beijing 100083, China

[c]Physical Chemistry and Soft Matter, Wageningen University and Research, Stippeneng, 4, 6708WE, Wageningen, the Netherlands

** Corresponding authors:*

E-mail: yuanli@cau.edu.cn; wenpch@126.com

This journal is © The Royal Society of Chemistry 2018

Contents

**1. Supplementary Experiment**	Page S3-S4
**Fig. S1, Fig. S2**	Page S4
**Fig. S3, Fig. S4**	Page S5
**Fig. S5, Fig. S6**	Page S6
**Fig. S7**	Page S7
**Supplementary References**	Page S7

This journal is © The Royal Society of Chemistry 2018

1 Supplementary Experiment

1.1 Scanning electron microscopy (SEM) analysis

The freeze-dried MC, theoretical DO30, DO50 and DO90 powder were adhered to the conductive adhesive of the SEM, and gold was sprayed. The prepared sample was subjected to a field emission SEM (JEOL, JSM 6301F) with a 5 mm working distance and an acceleration voltage of 5 kV.

1.2 Aqueous dispersibility analysis of the TEMPO oxidized CNFs

The same amounts of MC, theoretical DO30, DO50, and DO90 powder (5 mg) were dissolved in 5 mL ultra-pure water. After vortex gently, the dispersions were transferred to transparent glass bottles and photos were taken by a digital camera. The improvement of CNFs aqueous dispersibility was estimated by measuring the turbidity with an increasing CNFs concentration (10^{-5}, 10^{-4}, 10^{-3}, 10^{-2}, 10^{-1}, 1.0, 10 mg/mL) at 600 nm and 25 ^{o}C by UV-Vis spectrophotometer (UV-2450, Shimadzu, Japan). The turbidity (T) of the sample is given by

$$T = {}^{2.303A}\!/_{1} \qquad \text{(Eq. S1)}$$

where A is the observed absorbance and 1 is the pathlength of the cuvette [1].

1.3 Fourier infrared (FT-IR) spectra of TEMPO oxidized CNFs

Potassium bromide (200 mg) and 2 mg sample (MC and DO50, respectively) were mixed and grinded clockwise with a grinding rod. The mixture was pressed into a transparent tablet (thickness of ~0.5 mm) by an infrared tablet press at a pressure of 60 kN for 4 minutes. The MC and DO50 tablets were scanned by a Fourier Transform Infrared Spectrometer (Nicolet, Thermo Fisher Scientific) and resulting data were processed with Origin 8.0 software.

1.4 Size and zeta-potential of microcrystalline cellulose, DO50 and DO90 polymers

MC, DO50 and DO90 powders were dissolved in ultra-pure water with concentrations of 1 mg/mL, respectively. The size and zeta (ζ) potential was measured by malvern laser particle size analyzer (Malvern, Aetasizer Nano ZS). MC size distribution was monitored by Malvern Mastersizer 3000 (Malvern, UK) connected to a wet dispersion

unit (Hydro EV, Malvern, UK) and the data was recorded using the respective software (Mastersizer 3000, v1.0.1)[2].

1.5 ^{13}C Solid-NMR spectra

MC, DO50, DO90 and completely oxidized DO100 were measured by ^{13}C solid-state nuclear magnetic spectroscopy (Bruker, AVANCE III) to confirm the TEMPO oxidation occurred specifically at the C6 position and the introduction of the carboxyl group.

2 Fig. S1~S7

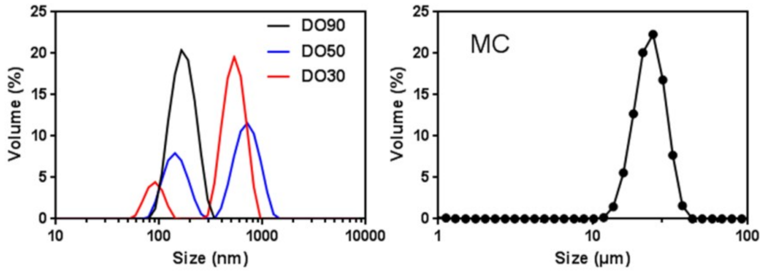

Fig. S1 Size distribution of DO30, DO50 and DO90 in nm and MC in μm. Measurement was performed at room temperature and the samples were diluted by deionized water.

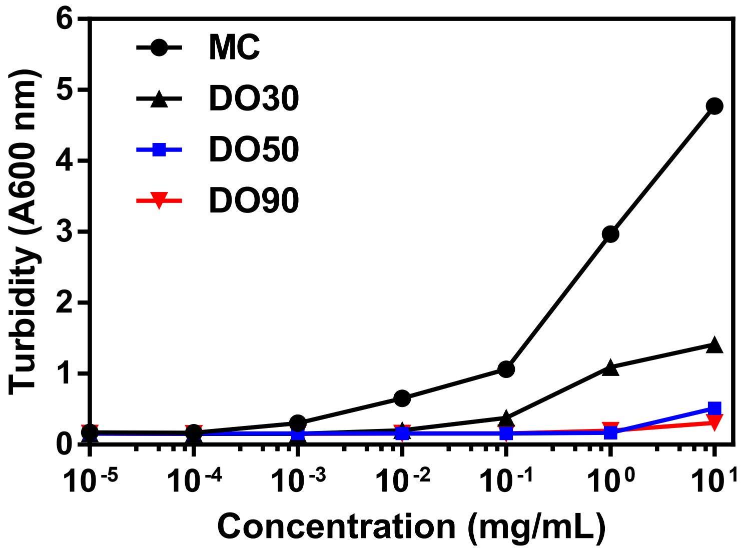

Fig. S2 Effect of concentrations (10^{-5}, 10^{-4}, 10^{-3}, 10^{-2}, 10^{-1}, 1.0, 10 mg/mL) on the turbidity of DO30, DO50, DO90 and MC in aqueous solution measured at excitation wavelength of 600 nm.

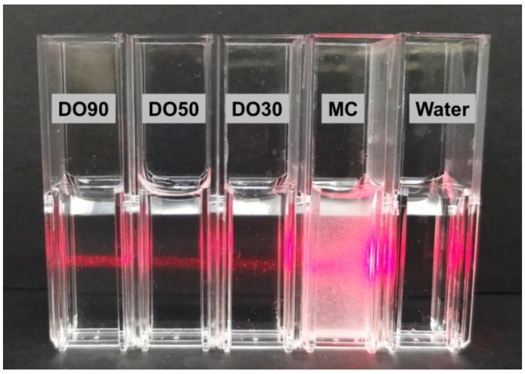

Fig. S3 Tyndall phenomenon of MC, DO30, DO50 and DO90. Water as blank control group.

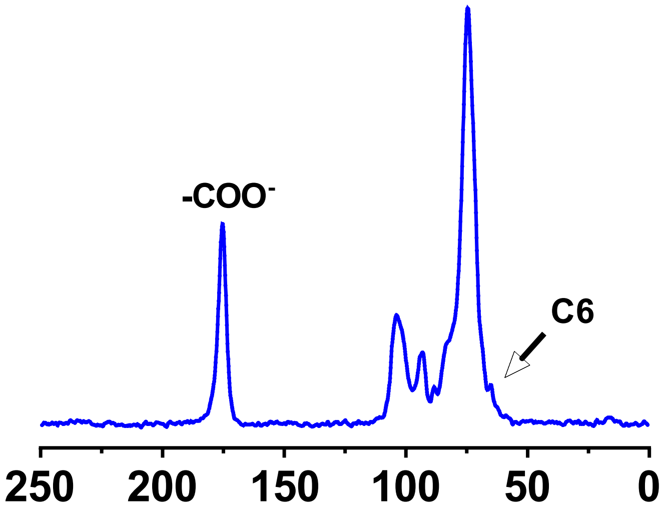

Fig. S4 The ^{13}C solid-state NMR spectrum of completely TEMPO oxidized cellulose DO100.

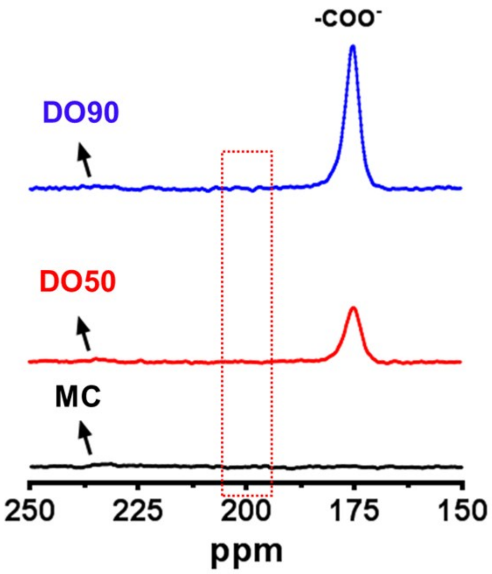

Fig. S5 The ^{13}C solid-state NMR spectrum of MC, DO50, and DO90 at 150-250 ppm representing C2 in the glucose unit. It indicated no ketones were produced in TEMPO-oxidation.

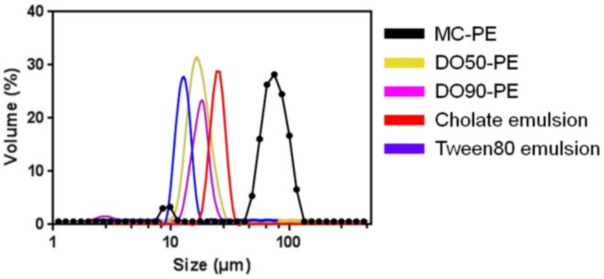

Fig. S6 Droplet size distribution of emulsions formed by MC, DO50, DO90, Cholate and Tween 80. Measurement was performed at room temperature and the emulsion was added to deionized water until an obscuration of approximately 10% was obtained.

This journal is © The Royal Society of Chemistry 2018

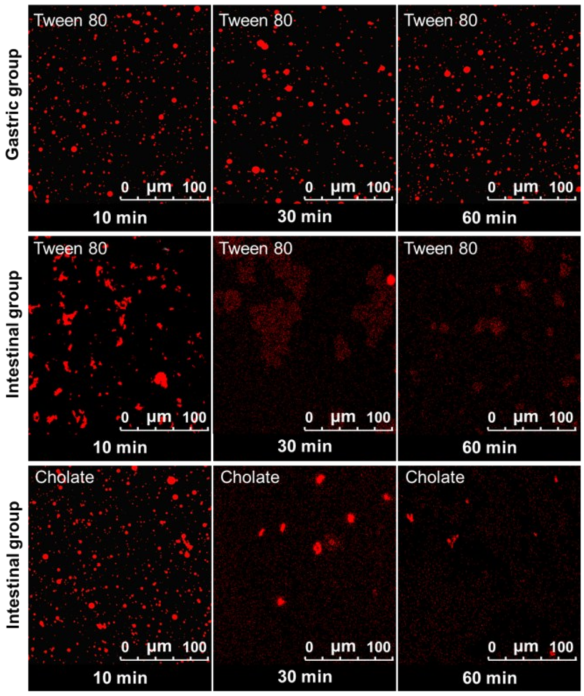

Fig. S7 CLSM images of Pickering emulsion formed by Tween 80 and Cholate digested in the simulated gastric (gastric group) and intestinal fluid (intestinal group) during 1 h. As a common sense, bile salts are involved in digestion and absorption in the small intestine, so we only examined the digestive experiments of Cholate formulation in the intestinal fluid. The oil phase was stained by Nile Red. The excitation and emission wavelength for Nile Red were 543 nm and 650 nm.

Supplementary References

1. K. N. Pearce and J. E. Kinsella, *J Agr Food Chem*, 1978, **26**, 716-723.
2. M. N. Hattrem, M. J. Dille, T. Seternes and K. I. Draget, *Food Hydrocolloid*, 2014, 37, 77-85.
